# Dynamic chemokine profiles in cervical mucus during healthy pregnancy: IP-10 and MIP-1β as potential biomarkers of gestational immune adaptation

**DOI:** 10.3389/fimmu.2026.1825948

**Published:** 2026-05-15

**Authors:** Ho Yen Chueh, Fang-Yu Chang, Tian Chi Tsai, Chia Lin Chang

**Affiliations:** 1 Department of Obstetrics and Gynecology, Chang Gung Memorial Hospital Linkou Medical Center, Taoyuan, Taiwan; 2 Chang Gung University School of Medicine, Chang Gung University, Taoyuan, Taiwan

**Keywords:** CCL4, cervical mucus, cervicovaginal, CXCL10, mucosal immunity, pH, pregnancy, reproductive immunology

## Abstract

**Objective:**

The cervicovaginal microenvironment, influenced by factors such as pH and cytokine profiles, plays a critical role in cervical remodeling, membrane rupture, and uterine contractions, key processes in maintaining pregnancy and triggering labor. Studies of key cytokines in cervical mucus during normal pregnancy may provide non-invasive indicators of pregnancy health.

**Methods:**

We conducted a prospective cohort study analyzing cervical mucus samples from 133 pregnant women across three gestational periods. pH was measured using colorimetric strips, and a panel of 14 cytokines and growth factors was quantified using multiplex immunoassay. Statistical analyses included Kruskal-Wallis tests for group comparisons and Pearson correlation coefficients to assess relationships between variables.

**Results:**

Significant changes were observed in cervical mucus pH and specific cytokines throughout pregnancy. IP-10 (CXCL10) and MIP-1β (CCL4) levels were markedly elevated in the first trimester and showed significant positive correlations with pH (r = 0.199, p = 0.022 for IP-10; r = 0.419, p < 0.0001 for MIP-1β). These associations persisted after adjustment for BMI in multivariable regression models (β = 110.99, p = 0.004 for IP-10; β = 55.97, p = 0.002 for MIP-1β), with medium-to-large effect sizes (Cohen’s d = 0.75–1.15), indicating that early-pregnancy levels were approximately double those of late pregnancy. These chemokines demonstrated dynamic changes that correlated with gestational age, while other cytokines remained relatively stable.

**Conclusion:**

The findings identify IP-10 and MIP-1β as candidate markers of gestational immune adaptation, with their correlation to pH suggesting a coordinated immunological response in the cervicovaginal environment during the transition from early to late pregnancy. These dynamic changes may offer new avenues for non-invasive assessment of pregnancy health and future investigation of adverse pregnancy outcomes.

## 1 Introduction

Cervical mucus plays a vital role in regulating fertility and reproductive health. Beyond its well-established use in fertility awareness methods (FAM) and assisted reproductive technology (ART), cervical mucus provides insight into the local immune environment of the reproductive tract (1–3). Its composition is influenced by hormonal regulation (e.g., estrogen levels), immune signaling, and infections, making it a candidate medium for assessing reproductive health and complications. Importantly, the cervical canal represents an immunologically distinct compartment from the lower vaginal tract, with a naturally higher pH, different microbial composition, and a unique interface with the uterine cavity (7, 28). Endocervical sampling therefore isolates the local cervical immune milieu more precisely than vaginal fornix sampling, which collects a mixture of secretions from multiple sources.

Previous research has examined the physical properties (e.g., viscosity, texture) and molecular composition of cervical mucus in the context of infection detection, sperm-mucus interactions, and infertility-related disorders such as endometriosis and endometritis (2, 4–6). Of note, cytokines present in cervical mucus have been associated with mucosal immunity, implantation, and protection against infections (7, 8).

During pregnancy, immune responses are tightly regulated and appear to shift from pro-inflammatory to anti-inflammatory to pro-inflammatory across trimesters (9). While a certain level of inflammation is a regular part of pregnancy, a dysregulated inflammatory response in the cervix can lead to cervical ripening and premature labor (10–13). Earlier studies have implicated IL-1 and IL-8, among others, in processes related to tissue remodeling and immune defense during pregnancy (14). However, studies on shifts in the cytokine profile in cervical mucus remain scarce. We hypothesized that analyzing cytokine and pH profiles in cervical mucus throughout gestation would reveal dynamic immune changes reflective of normal pregnancy physiology in the cervical tract.

## 2 Materials and methods

### 2.1 Study design and participants

This exploratory study employed a cross-sectional analysis of a prospectively collected cohort of healthy pregnant women. This study was conducted at Chang Gung Memorial Hospital, Linkou, Taiwan, with IRB approval (IRB No. 201802156B0A3) and informed consent. The sample size was based on patient availability during the recruitment window (November 2019 to October 2020); no formal *a priori* power calculation was performed, as this was a hypothesis-generating study. The unequal distribution across gestational groups (early pregnancy (<14 weeks), n = 17; mid-pregnancy (14–26 weeks), n = 13; late pregnancy (>26 weeks), n = 103) reflects the natural enrollment pattern at our prenatal clinic, where most patients present in the third trimester for routine visits. This pattern reflects standard prenatal care in Taiwan, where most patients are referred to tertiary centers in the third trimester for routine monitoring and screening. Although the group sizes are unbalanced, non-parametric methods (Kruskal-Wallis tests) were employed to minimize the impact of unequal variances and sample sizes. Sensitivity analyses were conducted to assess the robustness of the findings (see Statistical Analysis).

Inclusion criteria included singleton pregnancies without known infections, chronic inflammatory conditions, or complications. Women with histories of preterm birth, cervical incompetence, preterm premature rupture of membranes (PPROM), congenital malformation, or autoimmune disorders were excluded to minimize potential confounding effects on cytokine expression. All participants underwent standard prenatal clinical evaluation; women with signs or symptoms of vaginitis, cervicitis, or other genital tract infections were excluded. Systematic microbiological cultures for bacterial vaginosis were not performed.

### 2.2 Sample collection and pH measurement

Cervical mucus was collected using Libo specimen swab kits (Pro-322221.4, Emelca Bioscience, Taiwan) during routine outpatient prenatal visits, prior to the onset of labor. Swabs were inserted into the endocervical canal for 20 seconds, transferred to sterile PBS-containing tubes with protease inhibitors, and stored at −80 °C. pH was measured using HEALTH MATE™ vaginal pH strips (DFIcare, Korea), a colorimetric dipstick. The strip was applied for 20 seconds in the endocervical canal, and the pH value was recorded immediately against a colorimetric reference chart. All measurements were performed by trained research nurses. Cervical length was measured by the standard procedure.

### 2.3 Cytokine/chemokine and growth factor analysis

Cytokines were quantified using the MILLIPLEX Human Cytokine/Chemokine Magnetic Bead Panel (MM HCYTA-60K-24; EMD Millipore) on a Luminex 200 system. The panel included 14 factors: EGF, FGF-2, G-CSF, IL-1α, IL-1β, IL-6, IL-8, IL-10, PDGF-AB/BB, IP-10 (Interferon-gamma-inducible protein 10, CXCL10), MCP-1 (CCL2), MIP-1α (Macrophage inflammatory protein 1α, CCL3), MIP-1β (Macrophage inflammatory protein 1β, CCL4), and VEGF. Data was analyzed with xPONENT software. Cytokine concentrations below the lower limit of detection were reported as extrapolated values from the standard curve by the xPONENT software; no values were censored or imputed.

### 2.4 Statistical analysis

Data analysis was performed using SPSS v25. Descriptive statistics were calculated for demographic and clinical variables, and normality was assessed via the Shapiro-Wilk test. The Shapiro-Wilk test indicated that most cytokine distributions deviated significantly from normality (p < 0.05), motivating the use of non-parametric group comparisons. Kruskal-Wallis tests with Dunn’s *post hoc* comparisons with Bonferroni adjustment for multiple pairwise comparisons were used to evaluate group differences (early pregnancy, mid-pregnancy, late pregnancy). Pearson correlation assessed relationships among cytokines, pH, and cervical length. p < 0.05 was considered statistically significant. Log-transformation of cytokine values was considered but not applied, as the non-parametric methods used for group comparisons are distribution-free, and the concordance between Pearson and Spearman correlations confirmed that untransformed analyses are robust.

Because maternal BMI differed significantly across gestational groups (p = 0.019), multivariable linear regression models were fitted with IP-10 and MIP-1β as dependent variables and gestational group (dummy-coded with >26 weeks as reference) and BMI as covariates, to assess whether the trimester-related differences persisted after BMI adjustment. Partial Pearson correlations were also computed between pH and cytokine levels controlling for BMI. As sensitivity analyses, we repeated the Kruskal-Wallis comparisons after excluding outliers (defined as values exceeding 3 standard deviations from the group mean) and performed rank-based regression as a non-parametric robustness check. Effect sizes were quantified using epsilon-squared (ϵ²) for Kruskal-Wallis tests and Cohen’s d for pairwise comparisons.

Pearson correlation was used as the primary method given its widespread use and interpretability for continuous variables. Because cytokine concentrations are often right-skewed, Spearman rank correlations were also computed as a complementary analysis. In all cases, the Spearman results were consistent with or stronger than the Pearson results (e.g., partial Spearman r = 0.234 for pH–IP-10 and r = 0.442 for pH–MIP-1β, compared with partial Pearson r = 0.197 and r = 0.409, respectively), confirming that the choice of correlation method does not affect the conclusions. Both unadjusted and BMI-adjusted (partial) correlations are reported to distinguish the raw observed associations from confound-free estimates.

## 3 Results

The cohort consists of 133 pregnant women, stratified by gestational age at study entry: early pregnancy (<14 weeks, n = 17), mid-pregnancy (14–26 weeks, n = 13), and late pregnancy (>26 weeks, n = 103) (**Table 1**). The median gestational age at enrollment was 11 weeks (range 10–13) for the early pregnancy group, 26 weeks (range 17–26; 11/13 enrolled at 26 weeks) for the mid-pregnancy group, and 31 weeks (range 27–33) for the late pregnancy group. The three groups were well-matched across most demographic and clinical parameters, with maternal age averaging around 32–33 years across all groups (p = 0.656) and similar gravidity, parity, and nulliparity rates. However, maternal BMI differed significantly between groups (p = 0.019), with early pregnancy patients having the lowest (23.17 ± 3.89) and late pregnancy patients having the highest (25.73 ± 3.54). Pregnancy outcomes were comparable across groups, with gestational age at delivery around 38 weeks, birth weights exceeding 3100 g, and excellent Apgar scores (≥8.92 at 1 minute and ≥9.92 at 5 minutes). Cesarean section rates varied from 23% to 47%, but did not reach statistical significance (p = 0.134). Newborn intensive care admissions were infrequent across all groups, ranging from 0% to 12.6% (p = 0.244).

**Table 1:** Demographic and clinical characteristics of patient groups. All values are presented as mean ± standard deviation (SD).

Clinical characteristics	Gestational age <14 weeks (N=17)	Gestational age 14-26 weeks (N=13)	Gestational age >26 weeks (N=103)	*p*-value
Maternal age (yr)	32.53 ± 4.82	32.92 ± 5.44	33.44 ± 5.12	0.656
BMI	23.17 ± 3.89	24.97 ± 3.39	25.73 ± 3.54	0.019*
Gravida	2.06 ± 0.9	2.38 ± 0.77	2.29 ± 1.24	0.543
Parity	0.76 ± 0.56	0.92 ± 0.76	0.70 ± 0.75	0.455
Nulliparity (%)	29.41 (5/17)	30.77 (4/13)	45.63 (47/103)	0.369
Gestational week at delivery	38.24 ± 0.75	37.92 ± 1.26	38.37 ± 1.22	0.371
Cesarean section (%)	47.06 (8/17)	23.08 (3/13)	32.04 (33/103)	0.134
Newborn birth weight	3112.1 ± 333.5	3172.5 ± 298.8	3146.9 ± 390.0	0.605
Apgar score at 1 min	9.00 ± 0	8.92 ± 0.28	8.97 ± 0.21	0.426
Apgar score at 5 mins	10.00 ± 0	9.92 ± 0.28	9.97 ± 0.17	0.421
Newborn OBN or ICU admission (%)	0 (0/17)	7.69 (1/13)	12.62 (13/103)	0.244

All data are represented as mean ± SD.

*p<0.05 P-values were derived from the Kruskal-Wallis test. BMI, Body Mass Index.

**Figure 1:**
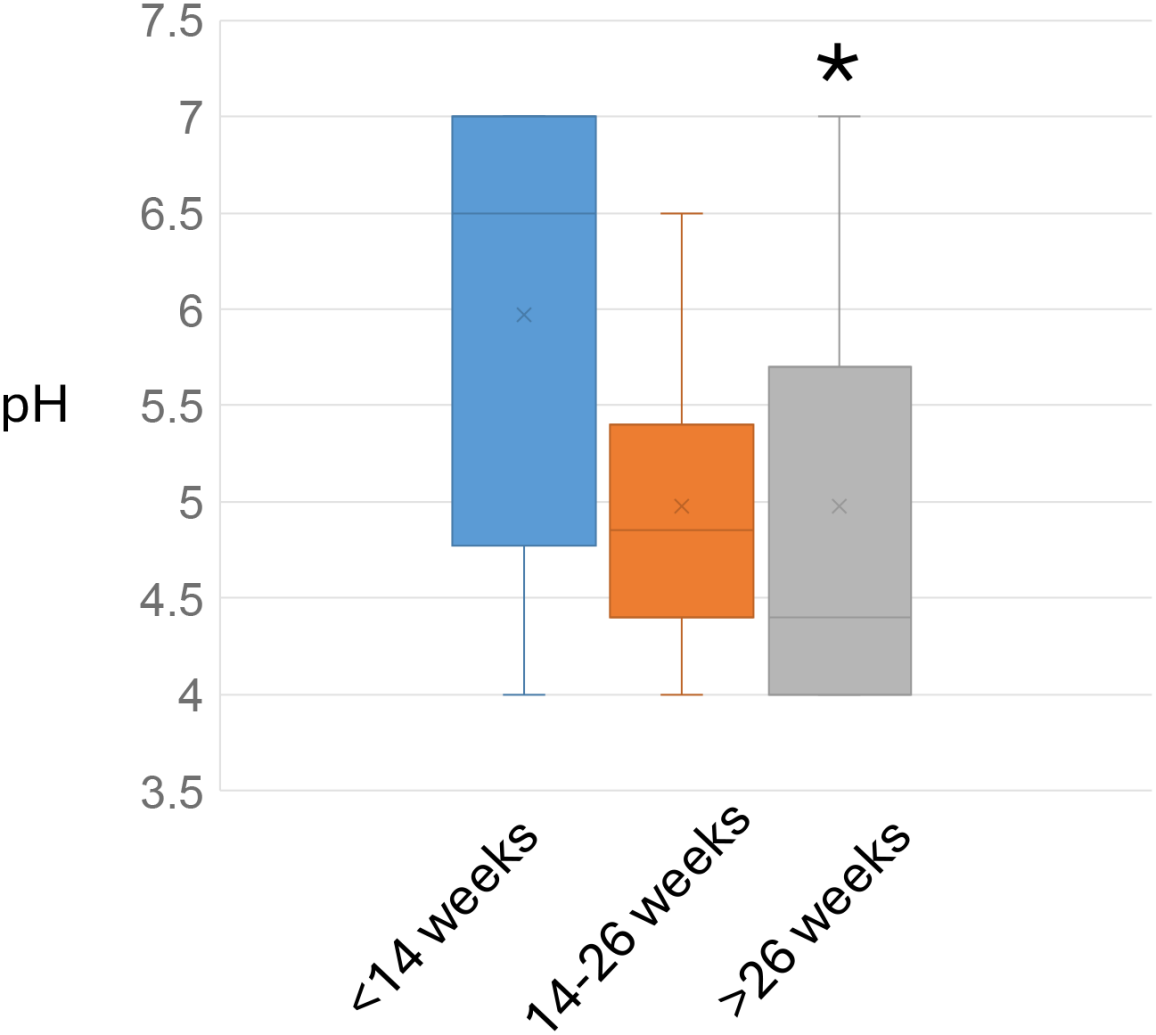
Cervical mucus pH levels across three gestational age groups. Box plots display the median (horizontal line), interquartile range (box), and 1.5×IQR whiskers (vertical lines). Data were compared using the Kruskal-Wallis test, followed by Dunn’s *post hoc* test for pairwise comparisons. The three gestational groups are: blue = early pregnancy (<14 weeks), orange = mid-pregnancy (14–26 weeks), and gray = late pregnancy (>26 weeks). Asterisks denote a statistically significant difference (*p < 0.05) compared to the early pregnancy group.

Among these patients, cervical length showed a modest decreasing trend across gestational groups: 4.09 ± 0.62 cm (early pregnancy), 4.04 ± 0.60 cm (mid-pregnancy), and 3.84 ± 0.76 cm (late pregnancy). The mucus pH showed a decreasing trend as gestation progressed, and the mucus pH in early pregnancy (5.97 ± 1.07) was significantly higher compared to that during late pregnancy (4.98 ± 1.09; p < 0.05) (**Figure 1**; **Table 2**).

**Table 2:** Cervical length, pH, and cytokine profiles of patient groups. The levels of cytokines and growth factors were presented as pg/ml.

Clinical variables	Gestational age <14 weeks (N=17)	Gestational age 14-26 weeks (N=13)	Gestational age >26 weeks (N=103)
Cervical length (cm)	4.09 ± 0.62	4.04 ± 0.60	3.84 ± 0.76
pH	5.97 ± 1.07	5.00 ± 0.73	4.98 ± 1.09 *
EGF	9.79 ± 9.82	6.66 ± 4.31	6.85 ± 10.08
FGF-2	39.27 ± 25.07	33.59 ± 10.00	30.79 ± 15.83
G-CSF	4559.90 ± 3423.92	4606.81 ± 4355.03	3906.67 ± 3044.68
IL-1α	73.88 ± 47.89	180.33 ± 205.29	133.76 ± 188.29
IL-1β	75.48 ± 64.42	303.90 ± 364.97	127.66 ± 193.44
IL-6	245.71 ± 335.05	145.33 ± 181.23	146.04 ± 180.74
IL-8	1488.74 ± 1418.62	1933.00 ± 1777.46	1588.64 ± 1427.05
IL-10	36.24 ± 42.36	20.94 ± 23.42	18.59 ± 30.75
IP-10	200.06 ± 188.11	43.35 ± 42.63 *	102.60 ± 134.46
MCP-1	242.98 ± 366.10	88.50 ± 56.26	198.84 ± 571.05
MIP-1α	56.71 ± 46.83	46.62 ± 67.16	42.78 ± 38.88
MIP-1β	104.25 ± 94.36	48.19 ± 75.12 *	47.77 ± 53.64 *
PDGF-AB/BB	38.88 ± 41.89	25.26 ± 20.75	35.92 ± 42.90
VEGF	102.12 ± 119.96	113.87 ± 111.97	112.70 ± 113.59

All data are represented as mean ± SD; cytokines/growth factors are shown in pg/ml.

*p<0.05; significantly different from the <14 weeks group. P-values were derived from the Kruskal-Wallis test. EGF, Epidermal Growth Factor; FGF-2, Fibroblast growth factor 2; G-CSF, Granulocyte Colony-Stimulating Factor; IL, Interleukin, IP-10, Interferon-gamma-inducible protein 10; MCP-1, Monocyte chemoattractant protein-1; MIP-1α, Macrophage inflammatory protein 1α; MIP-1β, Macrophage inflammatory protein 1β; PDGF, Platelet-derived growth factor; VEGF, Vascular endothelial growth factor. The N number of “pH data” in the early pregnancy (<14 weeks) group is 16, instead of 17.

**Figure 2:**
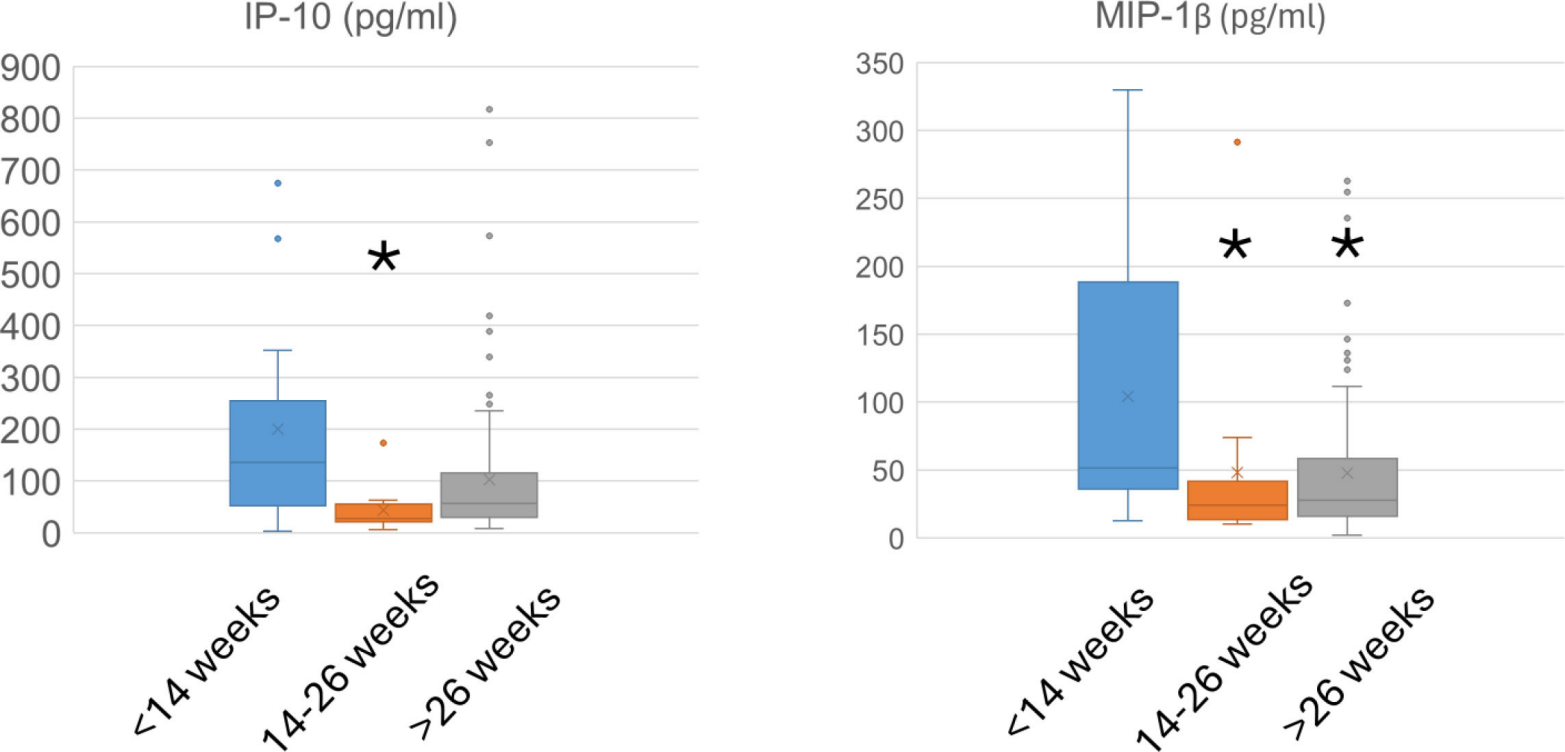
Cervical mucus IP-10 and MIP-1β levels (pg/ml) across three gestational age groups. Box plots display the median (horizontal line), interquartile range (box), and 1.5×IQR whiskers (vertical lines). Data were compared using the Kruskal-Wallis test, followed by Dunn’s *post hoc* test for pairwise comparisons. The three gestational groups are: blue = early pregnancy (<14 weeks), orange = mid-pregnancy (14–26 weeks), and gray = late pregnancy (>26 weeks). Asterisks denote a statistically significant difference (*p < 0.05) compared to the early pregnancy group.

Among the 14 cytokines and growth factors analyzed, only IP-10 and MIP-1β showed significant differences among the gestation groups (**Figure 2**; **Table 2**). Specifically, IP-10 levels were highest in early pregnancy, decreasing significantly in the second trimester. In contrast, MIP-1β levels were significantly elevated in early pregnancy compared to both the second and third trimesters (p < 0.05 for all). No significant changes were observed for IL-1α, IL-1β, IL-6, IL-8, IL-10, MCP-1, MIP-1α, EGF, FGF-2, G-CSF, PDGF-AB/BB, or VEGF across groups.

**Figure 3:**
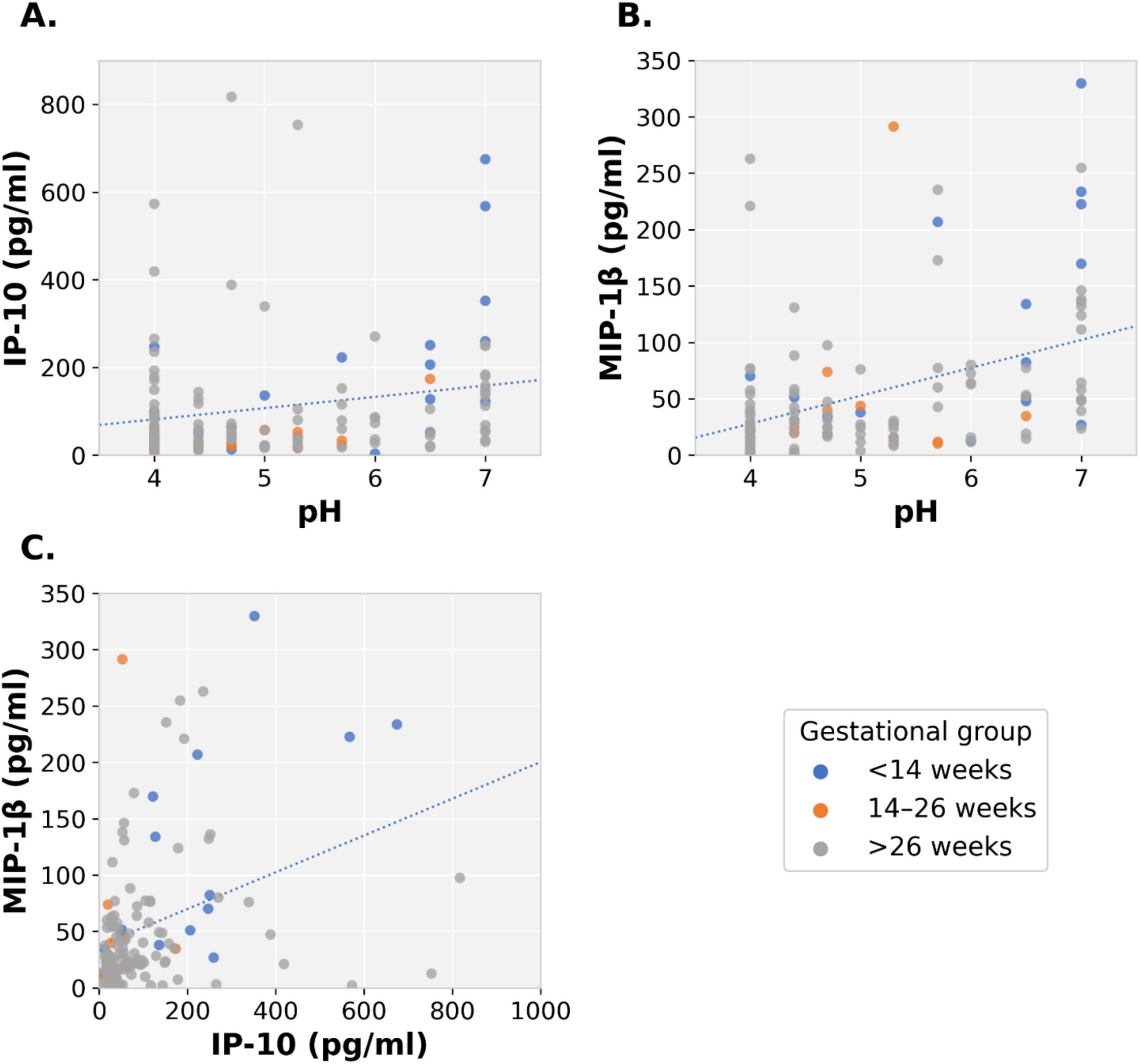
Correlations of cervical mucus pH, IP-10, and MIP-1β levels. Pearson correlation analysis showed that cervical mucus pH was positively correlated with IP-10 (A, r = 0.199) and MIP-1β (B, r = 0.419), and the two cytokines were positively correlated with each other (C, r = 0.356). Color-coded scatter plots showing correlations of cervical mucus pH, IP-10, and MIP-1β levels by gestational group (blue = early pregnancy (<14 weeks), orange = mid-pregnancy (14–26 weeks), gray = late pregnancy (>26 weeks). The dotted lines indicate the trend lines of the scatter plots. All correlations were computed across the entire cohort.

Correlation analyses were performed across the entire cohort (n = 132) to assess whether pH and chemokine levels co-varied across the gestational spectrum. Pearson correlation analysis showed a weak but statistically significant positive correlation between pH and IP-10 (r = 0.199, p = 0.022) and a moderate positive correlation with MIP-1β (r = 0.419, p < 0.001) (**Figures 3A, B**). IP-10 and MIP-1β were also significantly correlated (r = 0.356, p < 0.001; **Figure 3C**).

After adjustment for BMI in multivariable linear regression models, the associations between gestational group and both IP-10 (β = 110.99, 95% CI: 35.43 to 186.55, p = 0.004) and MIP-1β (β = 55.97, 95% CI: 21.64 to 90.30, p = 0.002) remained significant for the early versus late pregnancy comparison (**Supplementary Table 1**, **S2**). BMI itself was not a significant predictor of either IP-10 (p = 0.675) or MIP-1β (p = 0.352). While the Dunn’s *post-hoc* test identified the early pregnancy versus mid-pregnancy comparison as significant for IP-10, the BMI-adjusted regression identified the early pregnancy versus late pregnancy comparison as significant, reflecting the complementary sensitivity of non-parametric rank-based and parametric model-based approaches to group differences in the presence of high within-group variability.

Partial Pearson correlations between pH and IP-10 (r = 0.197, p = 0.023) and MIP-1β (r = 0.409, p < 0.001), controlling for BMI were consistent with the unadjusted analyses, confirming that BMI does not confound the pH–chemokine relationships (**Supplementary Table 3**). Similar results were obtained with the Spearman correlation analysis. Sensitivity analyses excluding outliers (>3 SD from group mean) yielded similar or stronger results (**Supplementary Tables 4**-**S8**). The effect size for the difference in IP-10 across gestational groups was medium (ϵ² = 0.093), with a large pairwise effect between early and mid-pregnancy (Cohen’s d = 1.15). For MIP-1β, the overall effect size was small-to-medium (ϵ² = 0.069), with a large pairwise effect between early and late pregnancy (d = 0.99). In clinical terms, the BMI-adjusted β coefficients indicate that early-pregnancy IP-10 and MIP-1β levels are approximately double those of late pregnancy (108% and 117% higher, respectively), underscoring that these are not merely statistically significant but biologically substantial differences.

## 4 Discussion

To our knowledge, this is the first study to systematically characterize the profiles of IP-10 (CXCL10) and MIP-1β (CCL4) in cervical mucus across gestational stages in a strictly healthy cohort with confirmed term delivery. Previous studies of cervical cytokines have predominantly focused on pathological pregnancies or high-risk populations, limiting our understanding of baseline immunological adaptations. Our identification of IP-10 and MIP-1β as dynamic immune markers that are significantly elevated in early pregnancy and positively correlated with cervical mucus pH provides novel evidence that the cervical mucus microenvironment in healthy pregnancy exhibits a distinct and targeted immunological signature in the first trimester, rather than the broad inflammatory changes previously assumed. These markers, in conjunction with pH, offer potential new avenues for monitoring gestational progression and the pathophysiology of pregnancy-related disorders.

IP-10 and MIP-1β are chemokines known to recruit immune cells, such as T cells and macrophages (15, 16). Prior work has linked IL-1 and IL-8, among other mediators, to tissue remodeling and immune defense during pregnancy (17, 18). These cytokines play a crucial role in orchestrating the complex interactions between the mother and the developing fetus, contributing to both immune defense and tissue remodeling during pregnancy (19–21). Elevated levels of IP-10 and MIP-1β in amniotic fluid have been associated with intra-amniotic infections and risk of preterm birth (22, 23). However, their presence in healthy early pregnancy may suggest their involvement in immunological adaptation during early gestation and represent normal immune activation that supports implantation.

Contrary to some reports suggesting a pro-inflammatory surge in late pregnancy involving IL-6 and IL-8 (5, 24), our data from a strictly healthy, term-delivery cohort showed stable levels of these cytokines throughout gestation. These data suggest that, in the absence of pathological stimuli, the cervical environment maintains stable, low-level expression of these canonical inflammatory mediators. This discrepancy may be multifactorial. First, many previous studies included high-risk populations or women who subsequently developed complications such as preterm birth, where an inflammatory state is expected (25). Second, differences in sample collection methods (e.g., sponge vs. lavage) and immunoassay platforms may affect cytokine quantification (26). While most cytokines remain stable throughout gestation, the selective modulation of IP-10 and MIP-1β suggests that these markers may serve as indicators of cervical immune status.

Our findings also corroborate prior studies indicating pH modulations across gestation. The cervical and vaginal environments are more alkaline early in pregnancy and become more acidic later, likely reflecting changes in microbial composition and the hormonal milieu (27). The positive correlation between higher pH and elevated chemokines in early pregnancy is a key finding. While a low vaginal pH (around 4.5) is protective, the cervical canal is naturally more alkaline, with pH values that can approach neutrality or even exceed it, especially in early pregnancy (27). Our data suggests that this relative alkalinity is not merely a passive feature but may be associated with the local immune milieu. While the cross-sectional design precludes causal inference, the observed association is consistent with a model in which the alkaline environment is permissive for specific immune signaling during early pregnancy. For instance, the activity of specific antimicrobial peptides and immune cells is pH-dependent (28, 29), and the higher pH in early pregnancy may facilitate the expression of particular cytokines observed in our study.

Although the overall R² values of the regression models were modest (0.085 for IP-10; 0.099 for MIP-1β), this is expected given the high inter-individual variability inherent in mucosal cytokine measurements. This level of explained variance is characteristic of mucosal cytokine research, where inter-individual biological variability is inherently high and standard deviations routinely approach or exceed group means, as also observed in cervicovaginal cytokine studies (32, 33). Despite the modest R² values of the regression models (~9–10%), the medium overall effect sizes for IP-10 (ϵ² = 0.093) and MIP-1β (ϵ² = 0.069), together with the large pairwise Cohen’s d values between early and later gestational stages (d = 0.75–1.15 for IP-10; d = 0.68–0.99 for MIP-1β) indicate clinically meaningful differences that warrant attention. Importantly, the absolute magnitude of the differences — with early-pregnancy IP-10 and MIP-1β levels approximately double those of late pregnancy — is arguably more clinically relevant than variance-based metrics, as it defines a clear threshold that could inform future biomarker cutoff development. Furthermore, sensitivity analyses excluding outliers (>3 SD from group means) resulted in approximately doubled R² values (from ~9% to ~19–20%) and strengthened β coefficients (133.54 and 66.38, respectively), indicating that the primary analysis provides conservative estimates of the true gestational-stage differences. Moreover, the observation that mid-pregnancy and late-pregnancy levels were virtually indistinguishable for both MIP-1β (d = 0.01) and pH (d = 0.02) further refines the biological interpretation: the primary immunological shift in cervical mucus occurs early in gestation, likely reflecting the pro-inflammatory milieu required for implantation and early placentation, after which the cervical environment stabilizes into a lower-chemokine, lower-pH state that is maintained through term. Taken together, these data reflect a consistent and clinically meaningful immunological shift, supported by multiple complementary analytical approaches, rather than a statistical artifact driven by outliers, sample imbalance, or BMI confounding.

The selective elevation of IP-10 and MIP-1β in the cervicovaginal mucosa during early pregnancy parallels chemokine-mediated immune surveillance mechanisms at other mucosal surfaces. In the intestinal mucosa, IP-10 is constitutively expressed at low levels and is rapidly induced in response to interferon-γ signaling, where it functions to recruit CXCR3-positive Th1 cells and NK cells to respond to microbial challenge (34, 35). Similarly, in the respiratory tract, IP-10 from bronchial epithelial cells establishes chemokine gradients essential for the trafficking of activated T cells into the airways during inflammatory conditions (15, 36). MIP-1β has likewise been implicated in mucosal immune cell recruitment in the gut and the lungs (35, 37). In both the gut and the respiratory tract, a distinguishing feature of healthy mucosal homeostasis is the presence of tightly regulated, low-level chemokine expression that facilitates immune surveillance without triggering overt inflammation—a pattern strikingly consistent with our finding that IP-10 and MIP-1β are selectively modulated in healthy pregnancy while broad pro-inflammatory mediators such as IL-6 and IL-8 remain stable. The cervicovaginal mucosa during pregnancy may thus employ a conserved mucosal strategy in which specific chemokine gradients recruit targeted immune cell populations to maintain the immunological environment necessary for fetal-maternal tolerance.

Several limitations of this study warrant consideration. First, the cross-sectional design compares different women at different gestational timepoints rather than tracking the same individuals longitudinally, which limits our ability to infer within-individual trajectories. The unbalanced group sizes, with a larger proportion of participants in late pregnancy (n = 103) compared to early (n = 17) and mid-pregnancy (n = 13), reflect clinical enrollment patterns but may reduce statistical power for detecting differences. To mitigate this, we employed non-parametric tests robust to unequal group sizes and performed sensitivity analyses adjusting for BMI, which differed significantly across groups. Second, pH was measured using colorimetric strips, which have lower precision than electrode-based pH meters and may be subject to inter-observer variability in color interpretation. However, all measurements were performed by trained research nurses following a standardized protocol with a manufacturer-provided colorimetric reference chart. Any measurement imprecision would add random noise, which would attenuate correlations toward zero rather than inflate them, making the observed pH–chemokine associations conservative estimates. Third, we did not analyze the cervicovaginal microbiome, a key factor influencing both local pH and cytokine expression (30, 31). The interplay between specific bacterial communities (e.g., Lactobacillus-dominated versus dysbiotic states) and the observed cytokine changes is a critical area for future investigation, particularly given recent evidence that vaginal microbiota composition modulates IP-10 and MIP-1β levels in the cervicovaginal environment and influences clinical outcomes (38). Nonetheless, the stability of canonical inflammatory markers (IL-6, IL-8, IL-1β) throughout gestation in our cohort is consistent with undetected bacterial vaginosis, which typically elevates these mediators (39). Fourth, our single-center Taiwanese cohort may limit generalizability to other populations with different genetic, ethnic, dietary, or environmental backgrounds (30). Fifth, although all samples were collected during routine outpatient prenatal visits prior to the onset of labor, we did not record whether participants subsequently underwent spontaneous or induced labor. Labor is known to acutely alter cervicovaginal cytokine profiles (19); however, because our samples were collected antepartum, the profiles reported here likely reflect the basal gestational immune environment rather than labor-associated inflammatory changes. Finally, although we excluded women with known complications, subclinical infections or inflammatory processes that were not detected at enrollment may have influenced our results.

Because the profiles of IP-10 and MIP-1β, in conjunction with biophysical parameters like pH, have not been systematically investigated in a healthy pregnant cohort, this represents a significant gap in our understanding of the baseline immunological adaptations of the cervix during a normal pregnancy. From a clinical perspective, the identification of IP-10 and MIP-1β as gestational-stage-specific cervical markers in healthy pregnancy establishes a reference framework against which pathological deviations could be measured. Future case-control studies comparing these profiles in women who develop preterm birth, chorioamnionitis, or preterm premature rupture of membranes against the healthy baseline described here may reveal early warning signals detectable through non-invasive cervical sampling. The correlation of these chemokines with pH raises the hypothesis that a simple pH measurement might eventually complement more detailed immunological profiling, although its clinical utility would require validation in prospective studies that include pathological pregnancies.

In addition to highlighting specific cytokines as sensitive markers of the evolving gestational immune environment in the reproductive tract, these findings suggest that previously reported broad pro-inflammatory cytokine changes during gestation may partly reflect cohort composition, including high-risk or complicated pregnancies, rather than normal gestational physiology alone. Future longitudinal studies integrating multi-omic data, including the microbiome and metabolome, are warranted to determine whether the gestational chemokine patterns identified here differ in women who develop pregnancy complications, and to evaluate whether these markers have predictive value for the early identification of at-risk pregnancies.

## Data Availability

The original contributions presented in the study are included in the article/**Supplementary Material**. Further inquiries can be directed to the corresponding author.

## References

[1] Moghissi KS . Sperm migration through the human cervix. In: Coutinho EM Fuchs F , editors.Physiology and genetics of reproduction. Plenum Press, New York (1974). p. 273–82.

[2] Quayle AJ . The innate and early immune response to pathogen challenge in the female genital tract and the pivotal role of epithelial cells. J Reprod Immunol. (2002) 57:61–79. doi:10.1016/S0165-0378(02)00019-0 12385834

[3] Kutteh WH Prince SJ Hammond KR Kutteh CC Mestecky J . Variations in immunoglobulins and IgA subclasses of human uterine cervical secretions around the time of ovulation. Clin Exp Immunol. (1996) 104:538–42. doi:10.1046/j.1365-2249.1996.36742.x. PMID: 9099941 PMC2200440

[4] Menge AC Medley NE Mangione CM Dietrich JW . The incidence and influence of antisperm antibodies in infertile human couples on sperm-cervical mucus interactions and subsequent fertility. Fertil Steril. (1982) 38:439–46. doi:10.1016/S0015-0282(16)46578-7 7117571

[5] Kutteh WH Moldoveanu Z Mestecky J . Mucosal immunity in the female reproductive tract: correlation of immunoglobulins, cytokines, and reproductive hormones in human cervical mucus around the time of ovulation. AIDS Res Hum Retroviruses. (1998) 14:S51–55. 9581884

[6] Katz DF . Human cervical mucus: research update. Am J Obstet Gynecol. (1991) 165:1984–6. doi:10.1016/S0002-9378(11)90559-6. PMID: 1755453

[7] Wira CR Patel MV Ghosh M Mukura L Fahey JV . Innate immunity in the human female reproductive tract: endocrine regulation of endogenous antimicrobial protection against HIV and other sexually transmitted infections. Am J Reprod Immunol. (2011) 65:196–211. doi:10.1111/j.1600-0897.2011.00970.x. PMID: 21294805 PMC3837338

[8] Fahey JV Schaefer TM Channon JY Wira CR . Secretion of cytokines and chemokines by polarized human epithelial cells from the female reproductive tract. Hum Reprod. (2005) 20:1439–46. doi:10.1093/humrep/deh806. PMID: 15734755

[9] Mor G Cardenas I . The immune system in pregnancy: a unique complexity. Am J Reprod Immunol. (2010) 63:425–33. doi:10.1111/j.1600-0897.2010.00836.x. PMID: 20367629 PMC3025805

[10] Challis JR Lye SJ Gibb W . Prostaglandins and parturition. Ann N Y Acad Sci. (1997) 828:254–67. doi:10.1111/j.1749-6632.1997.tb48546.x. PMID: 9329846

[11] Yellon SM . Immunobiology of cervix ripening. Front Immunol. (2020) 10:3156. doi:10.3389/fimmu.2019.03156. PMID: 32038651 PMC6993120

[12] Yellon SM Ebner CA Sugimoto Y . Parturition and recruitment of macrophages in cervix of mice lacking the prostaglandin F receptor. Biol Reprod. (2008) 78:438–44. doi:10.1095/biolreprod.107.063404. PMID: 18003949 PMC4237585

[13] Sugimoto Y Yamasaki A Segi E Tsuboi K Aze Y Nishimura T . Failure of parturition in mice lacking the prostaglandin F receptor. Science. (1997) 277:681–3. doi:10.1126/science.277.5326.681. PMID: 9235889

[14] Challis JR Lockwood CJ Myatt L Norman JE Strauss JF Petraglia F . Inflammation and pregnancy. Reprod Sci. (2009) 16:206–15. doi:10.1177/1933719108329095. PMID: 19208789

[15] Dufour JH Dziejman M Liu MT Leung JH Lane TE Luster AD . IFN-gamma-inducible protein 10 (IP-10; CXCL10)-deficient mice reveal a role for IP-10 in effector T cell generation and trafficking. J Immunol. (2002) 168:3195–204. doi:10.4049/jimmunol.168.7.3195. PMID: 11907072

[16] Schall TJ Bacon K Toy KJ Goeddel DV . Selective attraction of monocytes and T lymphocytes of the memory phenotype by cytokine RANTES. Nature. (1990) 347:669–71. doi:10.1038/347669a0. PMID: 1699135

[17] Romero R Dey SK Fisher SJ . Preterm labor: one syndrome, many causes. Science. (2014) 345:760–5. doi:10.1126/science.1251816. PMID: 25124429 PMC4191866

[18] Cappelletti M Della Bella S Ferrazzi E Mavilio D Divanovic S . Inflammation and preterm birth. J Leukoc Biol. (2016) 99:67–78. doi:10.1189/jlb.3MR0615-272RR. PMID: 26538528

[19] Gomez-Lopez N StLouis D Lehr MA Sanchez-Rodriguez EN Arenas-Hernandez M . Immune cells in term and preterm labor. Cell Mol Immunol. (2014) 11:571–81. doi:10.1038/cmi.2014.46. PMID: 24954221 PMC4220837

[20] Nadeau-Vallée M Obari D Palacios J Brien M Duval C Chemtob S . Sterile inflammation and pregnancy complications: a review. Reproduction. (2016) 152:R277–92. doi:10.1530/REP-16-0453. PMID: 27679863

[21] Shynlova O Lee YH Srikhajon K Lye SJ . Physiologic uterine inflammation and labor onset: integration of endocrine and mechanical signals. Reprod Sci. (2013) 20:154–67. doi:10.1177/1933719112446084. PMID: 22614625

[22] Romero R Chaemsaithong P Korzeniewski SJ Tarca AL Bhatti G Xu Z . Clinical chorioamnionitis at term II: the intra-amniotic inflammatory response. J Perinat Med. (2016) 44:5–22. doi:10.1515/jpm-2015-0045. PMID: 25938217 PMC5891100

[23] Romero R Gotsch F Pineles B Kusanovic JP . Inflammation in pregnancy: its roles in reproductive physiology, obstetrical complications, and fetal injury. Nutr Rev. (2007) 65:S194–202. doi:10.1111/j.1753-4887.2007.tb00362.x. PMID: 18240548

[24] Witkin SS Linhares IM Giraldo P . Bacterial flora of the female genital tract: function and immune regulation. Best Pract Res Clin Obstet Gynaecol. (2007) 21:347–54. doi:10.1016/j.bpobgyn.2006.12.004. PMID: 17215167

[25] Goldenberg RL Hauth JC Andrews WW . Intrauterine infection and preterm delivery. N Engl J Med. (2000) 342:1500–7. doi:10.1056/NEJM200005183422007. PMID: 10816189

[26] Kemp MW . Preterm birth, intrauterine infection, and fetal inflammation. Front Immunol. (2014) 5:574. doi:10.3389/fimmu.2014.00574. PMID: 25520716 PMC4249583

[27] Chen Y Bruning E Rubino J Eder SE . Role of female intimate hygiene in vulvovaginal health: Global hygiene practices and product usage. Womens Health (Lond). (2017) 13:58–67. doi:10.1177/1745505717731011. PMID: 28934912 PMC7789027

[28] Wira CR Fahey JV Sentman CL Pioli PA Shen L . Innate and adaptive immunity in female genital tract: cellular responses and interactions. Immunol Rev. (2005) 206:306–35. doi:10.1111/j.0105-2896.2005.00287.x. PMID: 16048557

[29] Hickey DK Patel MV Fahey JV Wira CR . Innate and adaptive immunity at mucosal surfaces of the female reproductive tract: stratification and integration of immune protection against the transmission of sexually transmitted infections. J Reprod Immunol. (2011) 88:185–94. doi:10.1016/j.jri.2011.01.005. PMID: 21353708 PMC3094911

[30] Ravel J Gajer P Abdo Z Schneider GM Koenig SS McCulle SL . Vaginal microbiome of reproductive-age women. Proc Natl Acad Sci USA. (2011) 108:4680–7. doi:10.1073/pnas.1002611107. PMID: 20534435 PMC3063603

[31] Gajer P Brotman RM Bai G Sakamoto J Schütte UM Zhong X . Temporal dynamics of the human vaginal microbiota. Sci Transl Med. (2012) 4:132ra52. doi:10.1126/scitranslmed.3003605. PMID: 22553250 PMC3722878

[32] Ashford K Chavan NR Wiggins AT Sayre MM McCubbin A Critchfield AS . Comparison of serum and cervical cytokine levels throughout pregnancy between preterm and term births. AJP Rep. (2018) 8:e113–20. doi:10.1055/s-0038-1656534. PMID: 29868246 PMC5980496

[33] Buxton MA Meraz-Cruz N Sánchez BN Foxman B Gronlund CJ Beltran-Montoya J . Repeated measures of cervicovaginal cytokines during healthy pregnancy: understanding “normal” inflammation to inform future screening. Am J Perinatol. (2020) 37:613–20. doi:10.1055/s-0039-1685491. PMID: 30978743 PMC7003200

[34] Zhao Q Kim T Pang J Sun W Yang X Wang J . A novel function of CXCL10 in mediating monocyte production of proinflammatory cytokines. J Leukoc Biol. (2017) 102:1271–80. doi:10.1189/jlb.5A0717-302. PMID: 28899907

[35] Kulkarni N Pathak M Lal G . Role of chemokine receptors and intestinal epithelial cells in the mucosal inflammation and tolerance. J Leukoc Biol. (2017) 101:377–94. doi:10.1189/jlb.1RU0716-327R. PMID: 27899415

[36] Liu M Guo S Hibbert JM Jain V Singh N Wilson NO . CXCL10/IP-10 in infectious diseases pathogenesis and potential therapeutic implications. Cytokine Growth Factor Rev. (2011) 22:121–30. doi:10.1016/j.cytogfr.2011.06.001. PMID: 21802343 PMC3203691

[37] Lillard JW Singh UP Boyaka PN Singh S Taub DD McGhee JR . MIP-1alpha and MIP-1beta differentially mediate mucosal and systemic adaptive immunity. Blood. (2003) 101:807–14. doi:10.1182/blood-2002-07-2305 12393512

[38] Usyk M Schlecht NF Pickering S Williams L Sollecito CC Gradissimo A . molBV reveals immune landscape of bacterial vaginosis and predicts human papillomavirus infection natural history. Nat Commun. (2022) 13:233. doi:10.1038/s41467-021-27628-3. PMID: 35017496 PMC8752746

[39] Anahtar MN Byrne EH Doherty KE Bowman BA Yamamoto HS Soumillon M . Cervicovaginal bacteria are a major modulator of host inflammatory responses in the female genital tract. Immunity. (2015) 42:965–76. doi:10.1016/j.immuni.2015.04.019. PMID: 25992865 PMC4461369

