# Supplementary material for: Dynamic chemokine profiles in cervical mucus during healthy pregnancy: IP-10 and MIP-1β as potential biomarkers of gestational immune adaptation
Source: Front Immunol. 2026 May 15;17:1825948. doi: 10.3389/fimmu.2026.1825948 (PMC13219046; doi:10.3389/fimmu.2026.1825948)
Supplement: Supplementary file 1 [file Table1.docx]

## Table S1. IP-10: Unadjusted and BMI-Adjusted Regression

| **Variable** | **β** | **SE** | **95% CI** | **p-value** |
| --- | --- | --- | --- | --- |
| Unadjusted model |  |  |  |  |
| <14 weeks vs >26 weeks | 106.75 | 36.72 | (34.10, 179.40) | 0.004 |
| 14–26 weeks vs >26 weeks | −59.25 | 40.22 | (−138.83, 20.32) | 0.143 |
| R² = 0.084 |  |  |  |  |
| BMI-adjusted model |  |  |  |  |
| <14 weeks vs >26 weeks | 110.99 | 38.19 | (35.43, 186.55) | 0.004 |
| 14–26 weeks vs >26 weeks | −58.04 | 40.45 | (−138.08, 22.00) | 0.154 |
| BMI (kg/m²) | 1.38 | 3.27 | (−5.10, 7.85) | 0.675 |
| R² = 0.085 |  |  |  |  |

## Table S2. MIP-1β: Unadjusted and BMI-Adjusted Regression

| **Variable** | **β** | **SE** | **95% CI** | **p-value** |
| --- | --- | --- | --- | --- |
| Unadjusted model |  |  |  |  |
| <14 weeks vs >26 weeks | 60.25 | 16.73 | (27.15, 93.34) | <0.001 |
| 14–26 weeks vs >26 weeks | 0.42 | 18.32 | (−35.83, 36.67) | 0.982 |
| R² = 0.093 |  |  |  |  |
| BMI-adjusted model |  |  |  |  |
| <14 weeks vs >26 weeks | 55.97 | 17.35 | (21.64, 90.30) | 0.002 |
| 14–26 weeks vs >26 weeks | −0.81 | 18.38 | (−37.17, 35.55) | 0.965 |
| BMI (kg/m²) | −1.39 | 1.49 | (−4.33, 1.55) | 0.352 |
| R² = 0.099 |  |  |  |  |

## Table S3. Unadjusted and BMI-Adjusted Correlations

| **Variable Pair** | **Unadjusted Pearson r** | **p** | **BMI-adj Pearson r** | **p** | **BMI-adj Spearman r** | **p** |
| --- | --- | --- | --- | --- | --- | --- |
| pH vs IP-10 | 0.199 | 0.022 | 0.197 | 0.023 | 0.234 | 0.007 |
| pH vs MIP-1β | 0.419 | <0.001 | 0.409 | <0.001 | 0.442 | <0.001 |
| IP-10 vs MIP-1β | 0.356 | <0.001 | 0.357 | <0.001 | 0.369 | <0.001 |

## Table S4. Kruskal-Wallis Results After Outlier Exclusion

| **Cytokine** | **Outliers removed** | **n (analysis)** | **Kruskal-Wallis H** | **p-value** |
| --- | --- | --- | --- | --- |
| IP-10 | 4 | 128 | 15.101 | <0.001 |
| MIP-1β | 5 | 127 | 11.744 | 0.003 |

*N =132 due to a missing pH data.*

## Table S5. Pairwise Comparisons After Outlier Exclusion (Mann-Whitney U)

| **Cytokine** | **<14 wk vs 14–26 wk** | **<14 wk vs >26 wk** | **14–26 wk vs >26 wk** |
| --- | --- | --- | --- |
| IP-10 | U=163.0, p=0.002 | U=1152.0, p=0.005 | U=325.0, p=0.010 |
| MIP-1β | U=159.0, p=0.004 | U=1189.5, p=0.001 | U=518.0, p=0.473 |

### Table S6. BMI-Adjusted Results Excluding Outliers

| **Cytokine** | **Variable** | **β** | **95% CI** | **p-value** |
| --- | --- | --- | --- | --- |
| IP-10 (n=128) | <14 weeks | 133.54 | (79.39, 187.68) | <0.001 |
|  | 14–26 weeks | −49.30 | (−108.65, 10.06) | 0.103 |
|  | BMI | 2.70 | (−1.93, 7.34) | 0.251 |
| MIP-1β (n=127) | <14 weeks | 66.38 | (40.21, 92.56) | <0.001 |
|  | 14–26 weeks | −12.37 | (−41.02, 16.29) | 0.395 |
|  | BMI | −0.57 | (−2.82, 1.69) | 0.620 |

.

## Table S7. Overall Effect Sizes (Kruskal-Wallis)

| **Variable** | **Kruskal-Wallis H** | **p-value** | **Epsilon-squared (ε²)** | **Eta-squared (η²)** | **Interpretation** |
| --- | --- | --- | --- | --- | --- |
| IP-10 | 12.237 | 0.002 | 0.093 | 0.079 | Medium |
| MIP-1β | 9.075 | 0.011 | 0.069 | 0.055 | Small–Medium |
| pH | 10.755 | 0.005 | 0.082 | 0.068 | Medium |

*Interpretation thresholds: <0.01 negligible, 0.01–0.06 small, 0.06–0.14 medium, ≥0.14 large.*

## Table S8. Pairwise Effect Sizes (Cohen’s d)

| **Variable** | **<14 wk vs 14–26 wk** | **<14 wk vs >26 wk** | **14–26 wk vs >26 wk** |
| --- | --- | --- | --- |
| IP-10 | d = 1.15 (large) | d = 0.75 (medium) | d = −0.46 (small) |
| MIP-1β | d = 0.68 (medium) | d = 0.99 (large) | d = 0.01 (negligible) |
| pH | d = 1.04 (large) | d = 0.91 (large) | d = 0.02 (negligible) |

*Cohen’s d thresholds: <0.2 negligible, 0.2–0.5 small, 0.5–0.8 medium, ≥0.8 large.*
